# Hearing loss as the initial manifestation of *Chlamydia psittaci* pneumonia in an older adult: A case report and literature review

**DOI:** 10.1016/j.idcr.2026.e02690

**Published:** 2026-07-16

**Authors:** Hailing Wu, Mengyuan Li, Yuan Zhang, Jinghua Zhang, Qiangui Liu

**Affiliations:** a Department of Respiratory Medicine, Beijing Geriatric Hospital, Beijing, China; b Department of Otolaryngology, Beijing Geriatric Hospital, Beijing, China

**Keywords:** *Chlamydia psittaci*, Pneumonia, Hearing loss, Older patient, Targeted next-generation sequencing

## Abstract

*Chlamydia psittaci* (*C. psittaci*) pneumonia is a zoonotic infection that typically presents with fever, cough, and fatigue. Sudden sensorineural hearing loss is an uncommon manifestation and may lead to early misdiagnosis. We report a 67-year-old woman admitted with fever for 1 week and hearing loss for 3 days. Chest computed tomography revealed extensive consolidation in the right lung, and empirical anti-infective therapy was ineffective. Targeted next-generation sequencing (tNGS) of bronchoalveolar lavage fluid (BALF) detected *C. psittaci*. Following confirmation of the diagnosis, antimicrobial therapy was switched to minocycline. The patient’s temperature normalized, hearing improved significantly, pulmonary lesions regressed, and follow-up chest imaging showed near-complete absorption of infiltrates. This case indicates that *C. psittaci* pneumonia can initially present with hearing loss. Careful evaluation of avian exposure history, characteristic imaging findings, and early molecular pathogen testing may facilitate timely diagnosis and tetracycline-based targeted treatment.

## Highlights

• Hearing loss can be the initial manifestation of *C. psittaci* pneumonia.
• Older patient.
• Avian exposure and lung consolidation should prompt suspicion of psittacosis.
• BALF tNGS enabled etiologic confirmation in this older patient.
• Minocycline improved fever, hearing loss, and pulmonary lesions.

## Introduction

Psittacosis is a zoonotic infection caused by *C. psittaci* that is commonly associated with exposure to infected birds or contaminated respiratory secretions and droppings [1]. Although the disease remains underrecognized, outbreak-based evidence indicates that psittacosis contributes to a clinically meaningful respiratory disease burden worldwide [2]. Recent multicenter and regional studies from China have shown that psittacosis is increasingly detected in patients with complicated or atypical pulmonary infection, particularly when metagenomic next-generation sequencing (mNGS) is used [3]. In humans, *C. psittaci* infection most commonly manifests as community-acquired pneumonia (CAP), and comparative studies of atypical-pathogen CAP indicate that patients with *C. psittaci* pneumonia often have prominent fever, fatigue, systemic inflammation, and extrapulmonary involvement [4]. Because routine culture is difficult and conventional tests may fail to detect the pathogen, mNGS or targeted next-generation sequencing (tNGS) of respiratory specimens, especially bronchoalveolar lavage fluid (BALF), has become an important method for etiologic confirmation and treatment guidance [5], [6], [7], [8]. However, uncommon extrapulmonary presentations, including hearing loss, remain easy to overlook [9], [10].

In the present case, an older patient had sudden hearing loss as a prominent early manifestation and initially sought care in an otolaryngology clinic. The final diagnosis of *C. psittaci* pneumonia was established by integrating the avian exposure history, chest imaging findings, and BALF tNGS results. This report summarizes the clinical features, diagnostic reasoning, and treatment course of the case and discusses recent clinical literature to support earlier recognition and more standardized management of similar patients.

## Case presentation

### Medical history

A 67-year-old woman was admitted on July 9, 2025, with "fever for 1 week and hearing loss for 3 days." One week before admission, she developed fever without an obvious trigger, with a maximum temperature of 39 °C, accompanied by chills, rigors, generalized myalgia, and fatigue. She occasionally coughed up a small amount of white sputum, and the fever was transiently relieved after self-administered antipyretics. Three days before admission, she developed apathy and hearing loss and visited an otolaryngology clinic at another hospital, where fever-related neurologic injury was considered. Pure-tone audiometry showed an average hearing threshold of 66 dB HL in the right ear, consistent with severe hearing loss, and 63 dB HL in the left ear, consistent with moderate hearing loss (Fig. 1A and B). Tympanometry showed type A curves in both ears (Fig. 2). She was diagnosed with bilateral sensorineural deafness and peripheral neuritis. She received oral Ginkgo biloba tablets, flunarizine hydrochloride, and mecobalamin, with poor response. Two days before admission, she developed recurrent fever, with a maximum temperature of 39.5 °C, accompanied by transient abdominal pain. Outpatient chest CT showed extensive patchy consolidation in the right lung with air bronchograms (Fig. 3A-D). Her medical history included hyperlipidemia, and she had brief contact with a parrot before symptom onset.

**Fig. 1:**
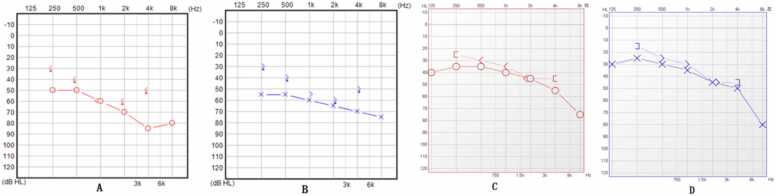
Pure-tone audiometry findings before and after treatment. A, right ear before treatment on July 9, 2025; B, left ear before treatment on July 9, 2025; C, right ear after treatment on July 21, 2025; D, left ear after treatment on July 21, 2025.

**Fig. 2:**
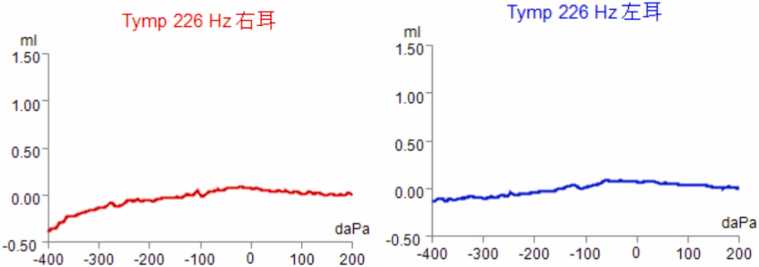
Tympanometry findings in the patient with Chlamydia psittaci pneumonia.

**Fig. 3:**
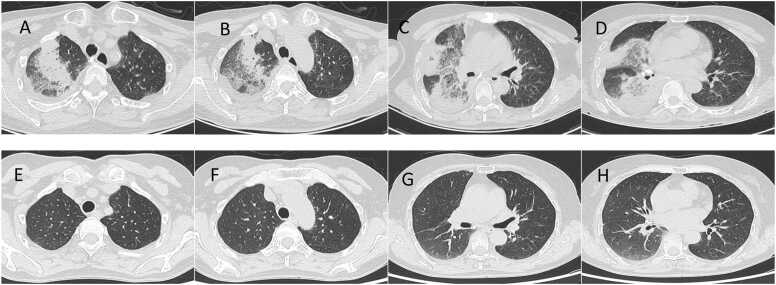
Chest computed tomography findings before and after treatment. A-D, chest CT on July 9, 2025, at admission, showing extensive patchy consolidation in the right lung with air bronchograms and a small right pleural effusion; E-H, follow-up chest CT on August 1, 2025, showing near-complete resolution of the pulmonary lesions.

On admission, physical examination showed a temperature of 39 °C, pulse of 89 beats/min, respiratory rate of 18 breaths/min, and blood pressure of 119/56 mmHg. The patient appeared apathetic and responded slowly. Bedside hearing screening suggested bilateral hearing impairment. Breath sounds were coarse bilaterally, and scattered moist rales were audible over the right lung. The abdomen was flat and soft, without tenderness or rebound tenderness.

Laboratory tests showed a white blood cell count of 8.15 × 10^9/L, neutrophil count of 7.16 × 10^9/L, lymphocyte count of 0.69 × 10^9/L, red blood cell count of 4.10 × 10^12/L, platelet count of 221 × 10^9/L, hemoglobin level of 126 g/L, neutrophil percentage of 87.9%, lymphocyte percentage of 8.5%, and C-reactive protein level of 247.93 mg/L. Chest CT revealed extensive patchy consolidation in the right lung with air bronchograms (Fig. 3A-D). Arterial blood gas analysis on room air showed pH 7.510, PaCO2 30.1 mmHg, and PaO2 55.9 mmHg. Alanine aminotransferase was 62.7 U/L, aspartate aminotransferase was 84.0 U/L, sodium was 131.5 mmol/L, chloride was 95.6 mmol/L, lactate dehydrogenase was 404 U/L, albumin was 26.9 g/L, interleukin−6 was 140.90 pg/mL, and procalcitonin was 0.90 ng/mL. Nasopharyngeal nucleic acid tests for influenza A, influenza B, severe acute respiratory syndrome coronavirus 2, *Mycoplasma pneumoniae*, and respiratory syncytial virus were negative, as were IgM antibody tests for *Chlamydia pneumoniae*, *Mycoplasma pneumoniae*, adenovirus, and parainfluenza virus. She was treated with nasal oxygen at 2 L/min and intravenous cefoperazone/sulbactam 3.0 g every 8 h plus moxifloxacin 0.4 g once daily.

Electronic bronchoscopy was performed after admission. A small amount of foamy secretion was observed in the bilateral bronchial lumina, and scattered punctate hemorrhage was seen in the bronchial mucosa at the right upper-lobe orifice and in the right middle-lobe bronchus. BALF was obtained from the right middle lobe and sent for tNGS, which detected *C. psittaci* (5886 ×10^3 copies/mL) and human herpesvirus 4 (Epstein-Barr virus, 61309 ×10^4 copies/mL). Based on the history of parrot exposure, clinical presentation, chest imaging features, and pathogen results, the final diagnoses were *C. psittaci* pneumonia, bilateral sensorineural hearing loss, type I respiratory failure, abnormal liver function, hyperlipidemia, hyponatremia, and hypochloremia. Antimicrobial therapy was changed to oral minocycline 100 mg twice daily. On day 2 after this adjustment, the peak body temperature decreased; on day 4, the temperature returned to normal, cough and sputum production improved, consciousness and hearing improved, and right-sided moist rales decreased.

On hospital day 9, her mental status had improved markedly, her responses had returned to normal, and she reported improved hearing. Repeat audiometry (Fig. 1C and D) showed an average hearing threshold of 40 dB HL in the right ear, consistent with moderate hearing loss, and 25 dB HL in the left ear, consistent with mild hearing loss, indicating marked recovery compared with pretreatment findings. Repeat laboratory testing showed a white blood cell count of 4.6 × 10^9/L, neutrophil count of 2.5 × 10^9/L, lymphocyte count of 1.62 × 10^9/L, red blood cell count of 3.62 × 10^12/L, platelet count of 320 × 10^9/L, hemoglobin level of 111 g/L, neutrophil percentage of 54.3%, lymphocyte percentage of 35.2%, and C-reactive protein level of 6.72 mg/L. Interleukin−6 decreased to 5.4 pg/mL, and procalcitonin was < 0.1 ng/mL. Liver function and electrolyte levels normalized. The patient was hospitalized for 13 days and continued oral minocycline after discharge for a total oral course of 10 days. At outpatient follow-up on August 1, 2025, chest CT (Fig. 3E-H) showed near-complete absorption of the pulmonary lesions, without obvious residual abnormalities.

## Discussion

While the overall incidence of *C. psittaci* pneumonia remains low, an increasing number of cases have been identified in recent years, largely attributable to advances in molecular diagnostic techniques for pathogen detection [3], [5], [6], [7], [8]. Accordingly, this pathogen should be included in the differential etiological diagnoses of CAP, especially for cases presenting with severe, atypical, or refractory pneumonia. This disease manifests with diverse clinical features. In addition to fever, cough, and fatigue, affected patients may also develop hypoxemia, liver function abnormalities, hypoalbuminemia, elevated inflammatory biomarkers, hyponatremia, and neurological symptoms [4], [11], [12], [13], [14]. The patient presented with high fever, extensive right lung consolidation, hypoxemia, elevated transaminases, and hypoalbuminemia. Notably, sudden sensorineural hearing loss stood out as the most prominent extrapulmonary manifestation. Such atypical clinical features may mask the primary respiratory infection and hinder early diagnosis.

Hearing loss is an uncommon manifestation of *C. psittaci* pneumonia. A recent case report combined with a literature review documented *C. psittaci*-associated pneumonia with hearing loss as the initial symptom, indicating that otological manifestations may precede or even predominate over respiratory symptoms [9], [10]. Compared with previously reported cases, the present case provides additional clinical insight by documenting an otolaryngology-first presentation with bilateral sensorineural hearing loss, only brief parrot exposure, extensive unilateral pulmonary consolidation, negative conventional respiratory pathogen tests, BALF tNGS confirmation, and objective audiometric improvement after tetracycline-based targeted therapy. These findings suggest that sudden hearing loss may be an early extrapulmonary clue to psittacosis pneumonia and that avian-exposure inquiry and pulmonary evaluation should be considered even when otological symptoms dominate the initial presentation.

The mechanism linking *C. psittaci* infection and hearing loss remains unclear and should be interpreted cautiously. Potential pathways include infection-triggered systemic inflammation or immune-mediated cochlear injury, fever- or hypoxemia-related microcirculatory disturbance affecting inner-ear perfusion, and possible inflammatory or neurotropic effects on the auditory pathway [10], [15], [16]. However, these proposed mechanisms are speculative in the present case. No inner-ear magnetic resonance imaging, cerebrospinal fluid analysis, vestibular testing, auditory brainstem response testing, or histopathological evidence was available to localize the lesion or demonstrate direct pathogen-related auditory injury. Therefore, the temporal association between pneumonia, hearing loss, and parallel improvement after minocycline supports a possible association but does not establish causality. The inference is further limited by the single-case design, and larger case series and mechanistic studies are needed to clarify whether *C. psittaci* infection can directly cause hearing impairment.

This case carries valuable clinical implications for clinical practice. The patient had a definite yet short-term history of parrot exposure. Chest CT revealed extensive patchy consolidation with air bronchograms in the right lung, accompanied by prominent inflammatory responses, lymphopenia, hypoxemia, elevated transaminases, and hyponatremia. These manifestations are consistent with findings from recent clinical studies, which have demonstrated that *C. psittaci* pneumonia is frequently characterized by fever, pulmonary consolidation, air bronchograms, pleural effusion, systemic inflammation, lymphopenia, hypoalbuminemia, and multiple organ dysfunction [6], [7], [11], [12], [13], [14]. Conventional respiratory pathogen tests yielded negative results, and empirical broad-spectrum antibiotic treatment showed no therapeutic effect. For patients with relevant epidemiological exposure history, severe or atypical imaging manifestations, and unsatisfactory response to conventional treatment, early implementation of BALF-based mNGS or tNGS is conducive to definitive etiological identification, shortening diagnostic time, and minimizing irrational empirical antimicrobial administration [5], [6], [7], [8].

With respect to treatment, recent clinical evidence indicates that timely etiological identification and early administration of tetracycline-based regimens are correlated with favorable clinical outcomes in patients with *Chlamydia psittaci* pneumonia [1], [6], [11]. In the present case, the initial empirical regimen consisting of cefoperazone/sulbactam combined with moxifloxacin yielded a poor therapeutic response. Following the identification of *C. psittaci* via BALF tNGS, the antimicrobial therapy was promptly switched to minocycline. The patient subsequently exhibited rapid defervescence, declined inflammatory biomarkers, gradual absorption of pulmonary lesions, and ameliorated hearing function. This clinical course further underscores the necessity of early pathogen-targeted therapy. Minocycline possesses favorable tissue penetration, rendering it a preferable option for oral sequential treatment and scenarios concerning drug accessibility. Although circulatory supportive care and neurotrophic interventions may facilitate the recovery of hearing impairment, such adjuvant treatments cannot substitute for pathogen-specific anti-infective therapy.

In conclusion, *C. psittaci* pneumonia in elderly adults can present with heterogeneous clinical profiles. While this infection generally manifests as atypical pneumonia, it may be concurrently complicated by rare otological or neurological sequelae [9], [10]. Clinicians should remain highly alert to potential *C. psittaci* infection in patients with avian exposure histories, persistent high fever, pulmonary consolidation, liver dysfunction, hyponatremia, hypoxemia, or hypoalbuminemia, as well as unexplained otological and neurological abnormalities [4], [11], [12], [13], [14]. For patients presenting with sudden sensorineural hearing loss or other uncommon extrapulmonary symptoms, meticulous epidemiological inquiry, multidisciplinary collaboration between otolaryngologists and pulmonologists, and repeated etiological evaluation are strongly recommended. Early clinical recognition, timely molecular pathogen detection, and prompt initiation of tetracycline-class antibiotics can optimize the management of pulmonary infection and facilitate the recovery of extrapulmonary organ dysfunction [5], [6], [7], [8].

## CRediT authorship contribution statement

**Wu Hailing:** Writing – review & editing, Writing – original draft, Conceptualization. **Zhang Yuan:** Investigation, Data curation. **LI Mengyuan:** Resources, Data curation. **Zhang Jinghua:** Formal analysis, Data curation. **Liu Qiangui:** Supervision, Resources.

## Ethics approval and consent to participate

The patient's legal guardians provided informed written consent for the publication of this clinical case report, with all data anonymized to protect confidentiality. This study was conducted in accordance with the ethical principles of the Declaration of Helsinki and followed the methodological recommendations of the CARE checklist for case reports.

## Declaration of Generative AI and AI-assisted technologies in the writing process

During the preparation of this work, the authors used OpenAI ChatGPT/Codex to assist with English language polishing and formatting. After using this tool, the authors reviewed and edited the content as needed and take full responsibility for the content of the publication.

## Funding

This work was supported by the Noncommunicable Chronic Diseases-National Science and Technology Major Project (2023ZD0506200/2023ZD0506201).

## Declaration of Competing Interest

The authors declare that they have no known competing financial interests or personal relationships that could have appeared to influence the work reported in this paper.

## Data Availability

Data sharing is not applicable to this article because no datasets were generated or analyzed during the current case report.
